# Mosaic structure of Mycobacterium bovis BCG genomes as a representation of phage sequences’ mobility

**DOI:** 10.1186/s12864-016-3355-1

**Published:** 2016-12-28

**Authors:** Olga L. Voronina, Marina S. Kunda, Ekaterina I. Aksenova, Andrey N. Semenov, Natalia N. Ryzhova, Vladimir G. Lunin, Alexandr L. Gintsburg

**Affiliations:** 0000 0000 9216 2496 grid.415738.c N.F. Gamaleya Federal Research Center for Epidemiology and Microbiology, Ministry of Health, Moscow, Russia

**Keywords:** *Mycobacterium bovis*, BCG, Daughter sub-strain, Phage, Genome rearrangement

## Abstract

**Background:**

The control of genome stability is relevant for the worldwide BCG vaccine preventing the acute forms of childhood tuberculosis. BCG sub-strains whole genome comparative analysis and revealing the triggers of sub-strains transition were the purpose of our investigation.

**Results:**

Whole genome sequencing of three BCG Russia seed lots (1963, 1982, 2006 years) confirmed the stability of vaccine sub-strain genome.

Comparative analysis of three *Mycobacteruim bovis* and nine *M. bovis* BCG genomes shown that differences between “early” and “late” sub-strains BCG genomes were associated with specific prophage profiles. Several prophages common to all BCG genomes included ORFs which were homologues to Caudovirales. Surprisingly very different prophage profiles characterized BCG Tice and BCG Montreal genomes. These prophages contained ORFs which were homologues to Herpesviruses. Phylogeny of strains cohort based on genome maps restriction analysis and whole genomes sequence data were in agreement with prophage profiles. Pair-wise alignment of unique BCG Tice and BCG Montreal prophage sequences and BCG Russia 368 genome demonstrated only similarity of fragmetary sequences that suggested the contribution of prophages in genome mosaic structure formation.

**Conclusions:**

Control of the extended sequences is important for genome with mosaic structure. Prophage search tools are effective instruments in this analysis.

**Electronic supplementary material:**

The online version of this article (doi:10.1186/s12864-016-3355-1) contains supplementary material, which is available to authorized users.

## Background

BCG (Bacille-Calmette-Guérin) vaccine is used broadly in various regions for the prevention of acute forms of childhood tuberculosis as part of the national childhood immunization programme. Despite these efforts, worldwide 9.6 million people are estimated to have fallen ill with TB in 2014. TB prevalence in 2015 was 42% lower than in 1990, as a result of vaccination against TB [1]. BCG vaccine is under control of the World Health Organization (WHO). The development of international requirements for the manufacture and control of BCG vaccine was first considered by the WHO Expert Committee on Biological Standardization (ECBS). In 2009 year WHO ECBS established WHO Reference Reagents for BCG vaccines of three different sub-strains (Danish1331, Tokyo 172-1 and Russian BCG-I) and quality control requirements, including genetic characterization of final lots and working seeds of BCG vaccines [2].

According to WHO and GMP requirements, BCG Russia sub-strain genome was sequenced in Russian research laboratories [3–5]. Now it is available in GenBank along with the sequences of other BCG sub-strains, because since the 1920s numerous sub-strains have evolved from the original strain BCG. And now *Mycobacterium bovis* BCG sub-strains comprise an excellent source for investigating the bacterial evolution. In this context, the endpoints of evolution are assessed likewise in the study of Darwinian biological species evolution [6]. Moreover, the progenitor of BCG strain was lost, a data describing sub-strain cultivation that could impact the sub-strain fitness are not available. Thus, genome characteristics can be used for searching a trigger of BCG sub-strains transition. Since phages are considered as an evolution tool, and prophages, as incomplete inserted phage sequences, contribute to the diversification of the bacterial genome architecture, the special emphasis was made on these mobile elements of bacterial genome.

Following Brussow H. et al. [7], 12 years later we can re-affirm that there is a renaissance of phage research. The information on whole bacterial genome collected in the international databases provides increasing resources for prophage sequences discovering. While discussing reintroduction of the fitness factor by phages, the researches usually refer to virulent factors of pathogenic bacteria [8]. Taking into account an essential role played by phages in the short-term adaptation processes, our goal was to assess a potential contribution of prophages in the mosaic structure of vaccine BCG sub-strains.

## Methods

### Bacterial strain

“368 shch” generation of sub-strain NSCPM 700001 *Mycobacterium bovis* BCG-I (Russia) from National State Collection of Pathogenic Microorganisms of “The Scientific Center for Expertise of Medical Application Products,” Russia’s Ministry of Health, was produced in 2006 and named ‘BCG Russia 368’. This generation is used for the production of the Russian BCG vaccine.

### Reference genomes

*M. bovis* complete genomes and one genome assembled in chromosome were imported from GenBank. They represented 11 biosamples. Three *M. bovis* strains were isolated from *Bos taurus*: AF2122/97 (Accession Number NC_002945.3); 1595 (NZ_CP012095.1); 30 (CP010332.1), one strain *M. bovis* BCG was isolated from a human patient with tuberculosis – 3281 (NZ_CP008744.1), and, the other strains were vaccine BCG sub-strains: Tokyo 172 (NC_012207.1), Pasteur 1173P2 (NC_008769.1), Moreau RDJ (NZ_AM412059.1), Mexico (NC_016804.1), Korea 1168P (NC_020245.2), 26/ATCC 35735/Montreal (CP010331.1), 63839/ATCC 35743/Tice (NZ_CP003494.1).

### DNA isolation

Preparation of genomic DNA for the whole genome sequencing was performed according to the Protocols [9]. DNA isolation for the whole genome mapping was made as described in [10].

### Genome sequencing and assembly

Two types of libraries were used for BCG Russia 368 genome sequencing by 454 Roche platform: shortgun and paired-end. The latter was built according to the 3 kb protocol. Sequencing procedure was performed using the GS Junior Titanium Sequencing Kit, i.e. GS Junior + Series XL+ Kit according to the manufacturer’s guidelines.

Assembly was performed with 454 Sequencing System Software v.2.7 and v.3.0 (Roche), yielding five scaffolds. PCR and Sanger sequencing were used for gap closure with primers presented in Additional file 1: Table S1. Most gaps were found in the sequences of PPE/PE-PGRS genes. Prediction of the secondary structures of this DNA sequences and calculation of the minimum free energy (MFE) structure were performed using RNAfold web server [11]. GC-reach DNA fragments amplification was optimized by both the involvement of 10% DMSO (Sigma) and 5% D-(+)-Trehalose dehydrate (Sigma). Sanger sequencing of amplicons was successful only in 5% DMSO presence.

### Whole genome map creating

Whole genome map (WGM) of the sub-strain BCG Russia 368 was created by the laboratory of OpGen Incorporated Company (Maryland, USA), according to the Argus™ Optical Mapping System User Manual [10]. DNA was digested with Nhel. Map Solver software version 3.2 was employed for creating the final circular WGM.

### Restriction maps analysis

GenBank files of the reference strains were taken for the restriction maps *in silico* generation with Nhel digestion. Map Solver software version 3.2 was utilized for the maps alignment and the cluster tree drawing. The lengths of tree branches indicated the relative differences between two nodes.

### Genome map analysis and visualization

*M. bovis* BCG Russia 368 genome map was performed in GeneWiz [12] and GenomeVx browsers [13, 14]. Genome atlas option of GeneWiz primarily GC Skew was selected as an appropriate instrument for verifying the accuracy of genome assemblies and OriC detection. Other DNA properties: Intrinsic Curvature, Stacking Energy, Position Preference, Global Direct repeats, Global Inverted repeats, AT content – were essential for genome structure description. GenomeVx browser was useful for the visualization of combination of different structural elements position.

### Genome annotation

The software Rapid Annotations Subsystems Technology and SEED [15, 16] were employed for annotating the genome BCG Russia 368. Complementary analysis was made with conserved domains search services: KEGG [17], KEGG OC [18], COGs [19]; protein subcellular localization prediction software: TMHMM Server v.2.0 [20], SignalP 4.1 Server [21], PSORTb version 3.0.2 [22], InterPro server [23, 24].

ISfinder was used for the prediction of insertion sequences (IS) elements and the additional annotation of resolvases, transposases and inregrases genes. IS elements family and sub-groups; as well as inverted repeats flanking IS elements were determined using ISfinder database [25, 26].

CRISPRfinder served as a tool for the prediction of clustered regularly interspaced short palindromic repeats and genes encoding CRISPR-associated Cas and Csm family proteins [27, 28].

The search for prophage sequences was made with PHAST (PHAge Search Tool) [29, 30]. GenVision Plug-In of the DNASTAR Lasergene programm package was selected for the visualization of prophages sequences.

### Phylogeny reconstruction

The full genome comparison and phylogeny reconstruction was based on BLAST alignment and Neighbor Joining algorithm [31] used in NCBI BLAST. The trees were represented by MEGA 6.0 [32].

BCG Russia 368 genome sequence was deposited in GenBank with the accession number NZ_CP009243.1.

## Results

### BCG Russia sub-strain genome stability

**Fig. 1:**
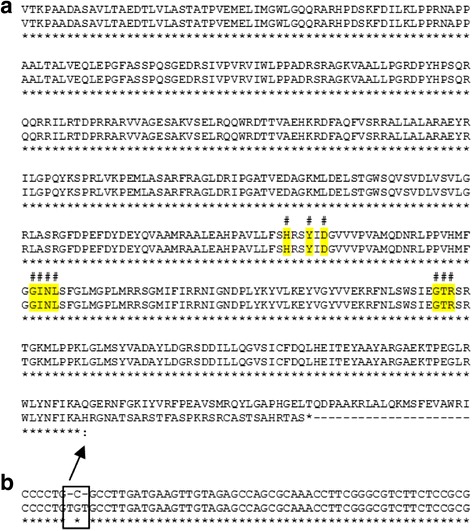
Comparison of whole and truncated variants of glycerol-3-phosphate acyltransferase in BCG Russia generations. **a** protein sequence. **b** sequence of gene fragment with mutation. Hash – amino acids residues important for the enzymatic activity

BCG sub-strain genome stability is the most important question of the vaccine manufacture. According to the WHO requirements for the production and control of BCG vaccines molecular genetic characterization should be included in the quality control of BCG vaccines. Thus, we made WGS of the last BCG Russia seed lot of 2006 named BCG Russia 368. Also, we sequenced two previous generations of BCG Russia sub-strain: BCG Russia 311 from seed lot of 1963 and BCG Russia 977 from seed lot 1982. Searching variants in DNA sequences demonstrated that SNP in the sub-strain BCG Russia 368 in position 3175301 according to the BCG Tokyo as reference, in uridylyltransferase gene, was a synonymous mutation without replacement in the protein sequence. However, this mutation was not present in the earlier generations. The second finding was an insertion of TGT instead of C in position 2744580, in glycerol-3-phosphate acyltransferase gene (Fig. 1). This mutation truncated the protein. It was shorter than original product, but contained the conservative domain and could be functional. Not all reads had this replacement: 14% in BCG Russia 311 and 54% in BCG Russia 977. It is important that this mutation was absent in the last generation (BCG Russia 368). These data could prove the stability of the sub-strain BCG Russia genome. The last generation genome BCG Russia 368 was deposited in GenBank with the Accession Number NZ_CP009243.1. In the text below we will discuss only the last generation of this sub-strain.

### Comparison of the “early” sub-strains genomes

BCG sub-strain Tokyo 172 genome, belonging to the “early” sub-strains group, was used as reference in the genome sequence comparison. First, BCG Tokyo is an “early” sub-strain closest to BCG Russia sub-strain as regards the time of its provision by the Pasteur Institute to Tokyo (in 1925). Second, as BCG Russia sub-strain it was lyophilized in 1940s and used later as a freeze-dried vaccine. Then, in 1960 the 172nd transfer on bile-potato medium was freeze-dried and adopted as a primary seed lot [33]. Finally, the genome of this seed lot was one of the first BCG genomes that were accurately sequenced, assembled and submitted to GenBank [34].

No substantial differences in genome sequences of BCG Russia 368 and BCG Tokyo 172 were found. The identified genomic differences were presented in Table 1 and could be subdivided into three groups: RD (region of differences), ins/del and SNP (Additional file 2: Table S2). Only two RD were detected between the “early” sub-strains. The first one was a 22 bp insertion in TetR family transcriptional regulator gene of BCG Russia 368 genome. This deletion (RD16) in BCG Tokyo genome was found in one variant of Japan BCG vaccine (Type I), submitted in GenBank. Type II had an RD16 band identical to those of other BCG sub-strains [35].

**Table 1:** Genomic differences of two pairs of sub-strains

Differences	Number of differences	
	BCG Russia368/BCG Tokyo 172	BCG Tokyo 172/Pasteur 1173P2 [34]
RD (more than 20 bp)	2	20
Ins/del <20 bp (1–9 bp)	10	20
SNP	52	68
*intergenic*	11	
*synonymous*	8	
*nonsynonymous (without nonsence)*	31	
*Nonsense as variant of nonsynonymous*	2	

The second RD was a 1602 bp deletion in BCG Russia 368 genome, corresponding to the region from 4110452 to 4112053 bp in BCG Tokyo 172, beginning in JTY_RS19265 (ribonuclease gene), including JTY_RS19270 (antitoxin VapB48 gene) and finishing inside JTY_RS19275 (glutamate-cysteine ligase gene).

The sub-strain BCG Russia was a progenitor of the sub-strains used for vaccine production in Bulgaria (BCG Sofia) and India. Nowadays, these BCG vaccines are among four variants used by the UNICEF (The United Nations Children’s Fund) on behalf of the Global Alliance for Vaccines and Immunization: BCG-Denmark produced by the Statens Serum Institute in Denmark, BCG-Russia (genetically identical to BCG-Bulgaria) produced by Bulbio (BBNCIPD) in Bulgaria and by the Serum Institute in India, and BCG-Japan produced by the Japan BCG Laboratory [36].

Based on the published data we could trace the genome characteristics of BCG Russia daughter sub-strains. The BCG sub-strain used for production in Bulgaria (named Sofia SL222) was analyzed by Stefanova T. et al. with *M. tuberculosis* microarrays. 1.6-kb deletion that affects the Rv3697c and Rv3698 homologues was detected. The authors also noted the deletion of this region in BCG Russia but not in any other strain [37]. It is concluded that RD 1602 bp is an old deletion, because BCG Pasteur was replaced with BCG Russia in Bulgaria BCG Laboratory in 1950s.

It should be noted that differences between the “early” sub-strain Tokyo and the “late” sub-strain Pasteur were more significant and the number of RD increased tenfold, according to Seki M. et al. [34].

Only ten ins/del (1–9 bp) differences were found between BCG Russia and BCG Tokyo genomes. Their number and size were lower than the ins/del differences between BCG Tokyo and BCG Pasteur genomes [34].

However, the number of SNPs was nearly the same in the two pairs of the genomes. Sixty percent of the SNP in BCG Russia 368 genome were nonsynonymous, but most of them were associated with conservative substitutions in the proteins. Only seven proteins had radical substitutions (Additional file 1: Table S2), though three of them were from PE-PGRS/PPE family. This finding has emphasized the significance of these proteins for BCG sub-strains adaptation.

### Restriction analysis of genome maps

**Fig. 2:**
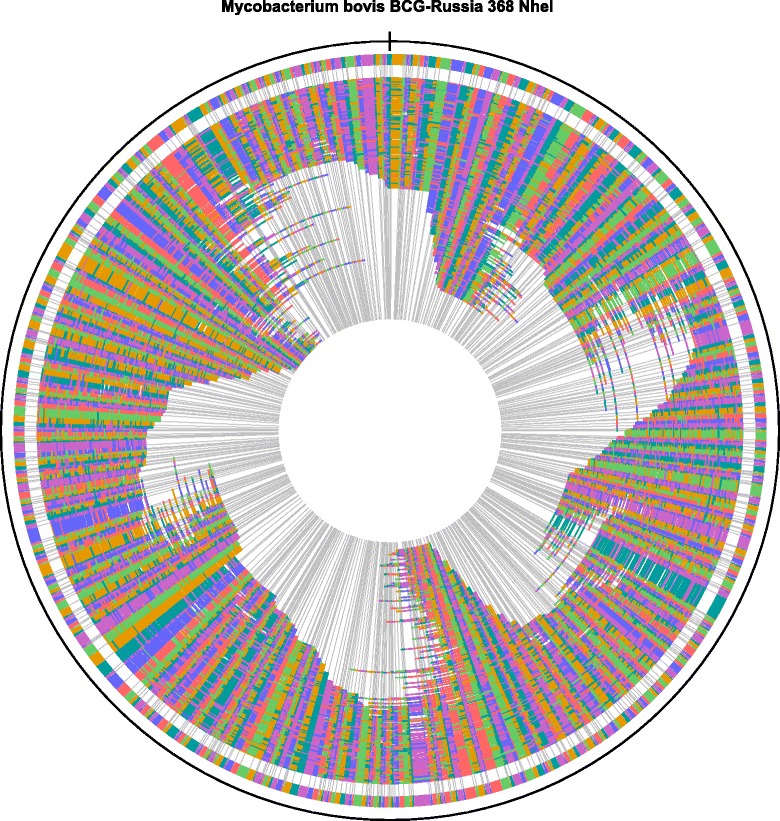
Circular restriction map of BCG Russia 368 whole-genome

**Fig. 3:**
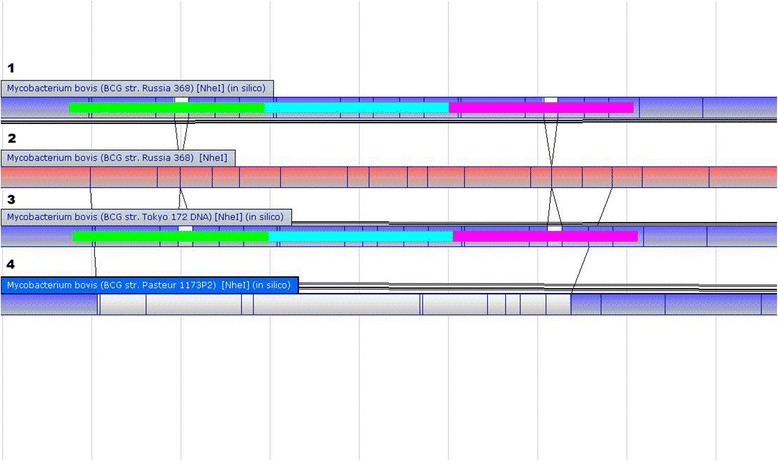
DU2 region of aligned optical maps for BCG Russia 368 and reference BCG sub-strains. **1** fragment of BCG Russia 368 optical restriction map created *in silico*. **2** whole-genome restriction map fragment of BCG Russia 368. **3** and **4** fragments of BCG Tokyo 172 and BCG Pasteur 1173P2 optical restriction maps created *in silico*. All restriction maps were obtained by DNA digestion with *NheI*. Restriction sites are shown as *vertical lines. Green*, *blue* and *purple* bars represents tree copies of DU2 region in BCG Russia 368 and BCG Tokyo 172 genomes. DU2 region includes the genes from the *astB* to the *sdhD* (*green bar*). In the copied regions (*blue* and *purple bars*), the *astB* gene was truncated

Genome comparison demonstrated that RD has played a more important role in BCG sub-strains differentiation. Method and equipment for performing this analysis were offered by OpGen Incorporated Company. As the first step, the assembling of DU2 region in BCG Russia 368 genome and the number of tandem duplications in this region were tested with the Argus™ Optical Mapping System. The WGM of BCG Russia 368 sub-strain is shown in Fig. 2. The comparison of DU2 regions was made separately (see Fig. 3). As you can see, the restriction maps of BCG Russia 368 and BCG Tokyo 172 are identical in this region, but differ from BCG Pasteur optical map. These data suggest the presence of triple tandem duplications in DU2 region of BCG Russia 368.

**Fig. 4:**
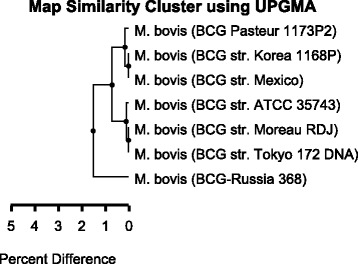
Map similarity cluster of BCG sub-strains with UPGMA. The cluster was obtained using created *in silico* optical restriction maps of BCG Russia 368 and six reference BCG sub-strains. OpGen MapSolver v.3.2.0. program with cluster method UPGMA and alignment score 3 was used for the cluster construction

The optical maps of six reference BCG sub-strains were added to the map similarity cluster construction (Fig. 4). The cluster was subdivided into two groups. BCG Tice (ATCC 35743) was included in the group of the “early” sub-strains, while BCG Mexico was added to the “late” sub-strains in accordance with the Nhel restriction fragments.

### Genome map analysis

**Fig. 5:**
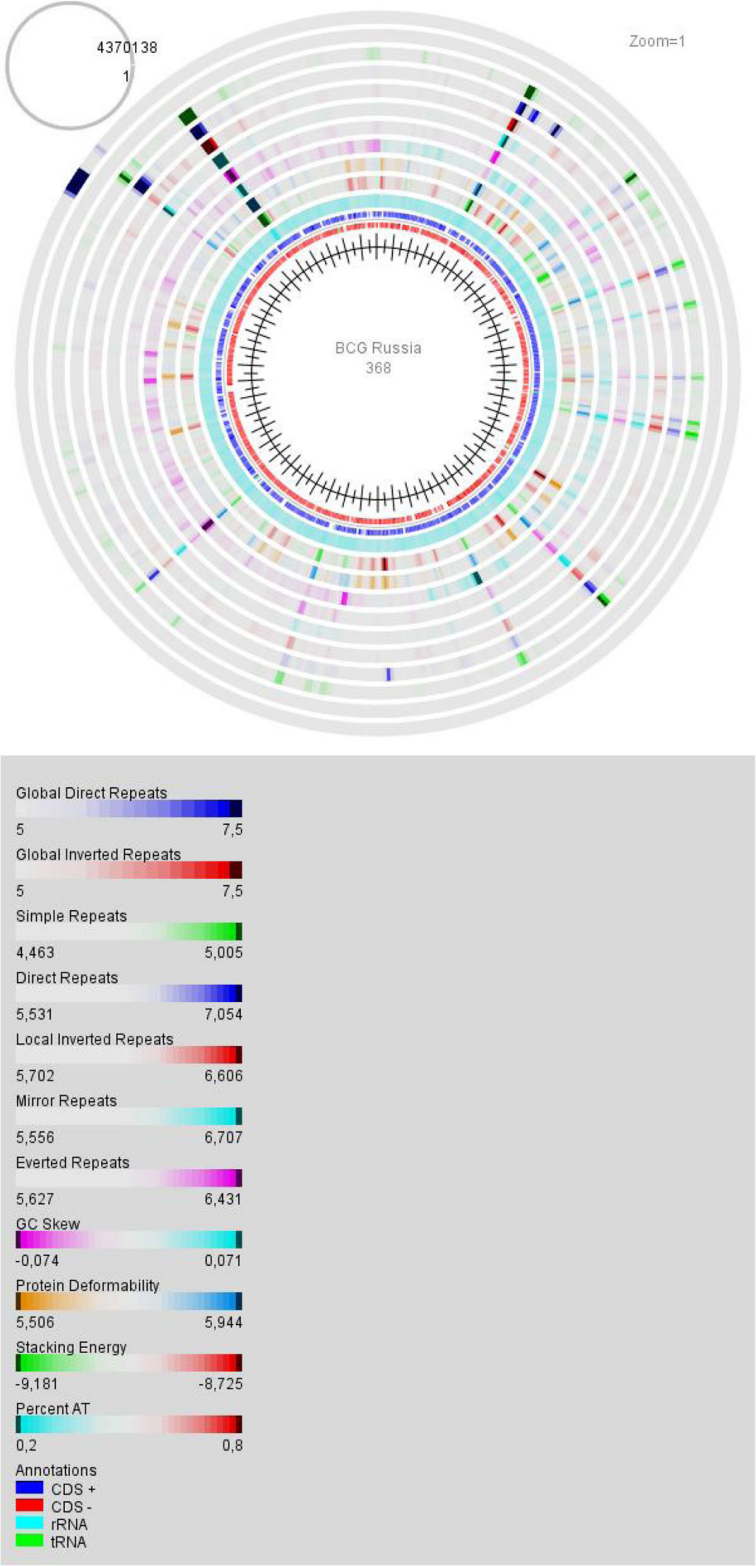
M. bovis BCG Russia 368 genome map

**Fig. 6:**
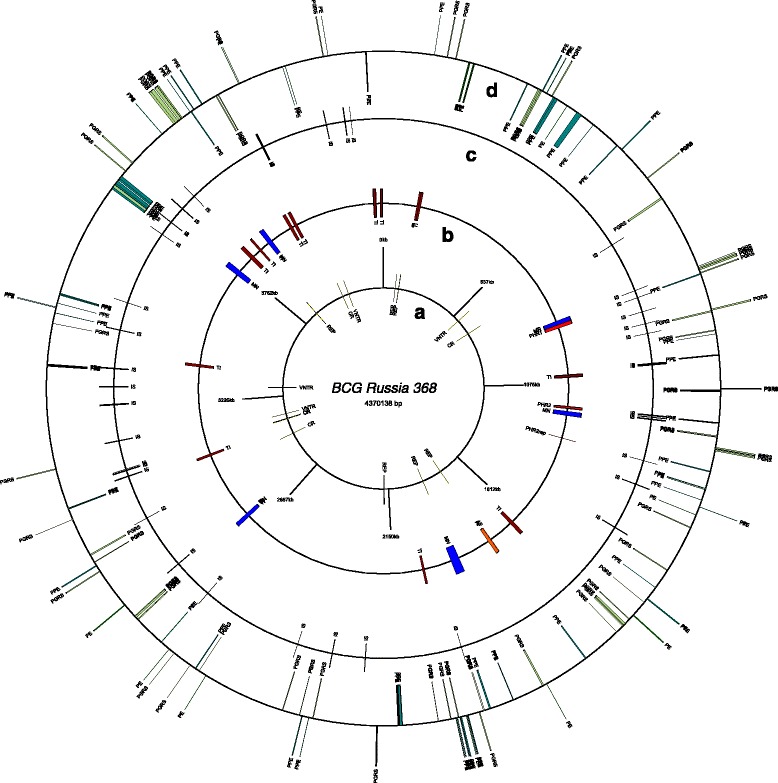
IS, repeats, prophage sequences and PE, PPE and PE_PGRS genes in BCG Russia 368 genome. The circular map of BCG Russia 368 genome was visualized using GenomeVx program. All prophage sequences were predicted by PHAST. Circles description: **a** repeats (REP, VNTR and CRISPR elements); **b** phages sequences (according to PHAST); **c** Insertion sequence elements; **d** genes for PE, PPE and PE_PGRS proteins. Abbreviations: IS - insertion sequence elements, REP - repetitive extragenic palindrome element; CR - CRISPR or possible CRISPR sequences predicted by CRISPRfinder; VNTR - variable number tandem repeat; TI - BCG Tice (CP003494.1) phages sequences; MN - BCG Montreal (CP010331.1) phages sequences; AF - *M. bovis* AF2122/97 (BX248333.1) phages sequences; PHR-2-rep - 922 bp repeat of 7.5 kb BCG Russia phages sequence; PHR-1–11 kb BCG Russia phages sequence; PHR-2–7.5 kb BCG Russia phages sequence. Bars colors indicated phages sequences discovered in different *M. bovis* genomes: *purple* - BCG Tice (CP003494.1); *blue* - BCG Montreal (CP010331.1); orange - *M. bovis* AF2122/97 (BX248333.1), *red* - BCG Russia 368 (CP009243)

After the verification of all tandem repeats and the gap closure, the sequence of assembling genome BCG Russia 368 was visualized in GeneWiz (Fig. 5). The place of the change in GC-Skew agreed with the OriC and the first nucleotide position in BCG Russia 368 genome. Different types of repeats, shown in this Figure, correlated with the specific genome elements identified with the specific resources (see “Methods”, Table 2). The position of these elements in BCG Russia 368 genome was marked on circles in Fig. 6 (Additional file 3: Table S3). As demonstrated, most of these elements are coinciding, overlapping or interconnecting. Therefore, the BCG genome can be described as repeat of repeats. Even prophage fragments have repeats in the genome sequence. However, while characterizing PE/PGRS genes, sometimes it is impossible to differentiate bacterial and phage genes.

**Table 2:** Specific genome elements in BCG Russia genome

Name	Number
REP	6
CRISPR	5
Prophage	2
PPE protein gene	66
PE protein gene	33
PE_PGRS protein gene	69
IS element	41

### Prophages in *M. bovis* genomes

**Fig. 7:**
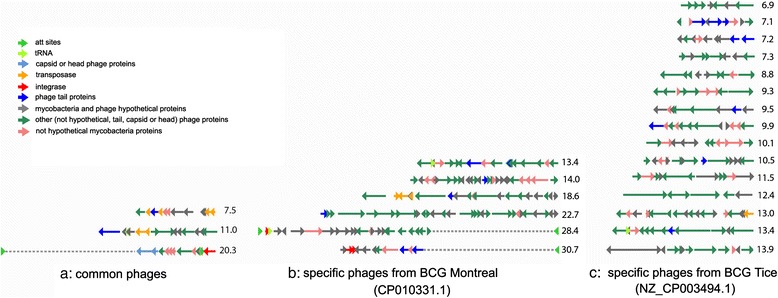
Phage types in Mycobacterium bovis genomes. *Color arrows* indicate the direction of phage genes. **a** common phages: 7,5, 11,0 and 20,3 kb – in all the analyzed strains (accession NZ_CP009243.1, NC_012207.1, NC_008769.1, NZ_CP003494.1, CP010331.1, NC_016804.1, NC_002945.3, NZ_AM412059.1, NZ_CP008744.1, NZ_CP012095.1, CP010332.1, NC_020245.2); **b** specific phages from BCG Montreal: 13,4, 14,0 18,6, 22,7, 28,4 and 30,7 kb; **c** specific phages from BCG Tice: 6,9, 7,1, 7,2, 7,3, 8,8, 9,3, 9,5, 9,9, 10,1 10,5, 11,5, 12,4, 13,0, 13,4 and 13,9 kb

Phages seem to be the best candidates for indentifying mosaic genome structure in the regions of repeats. We used PHAST for the computational identification of prophages in *M. bovis* genome sequences. All the discovered prophages (see Fig. 7) were split into three groups. The first group included the common prophages of *M. bovis* and *M. bovis* BCG. A 7.5 kb prophage was found in three *M. bovis* genomes and in the most of BCG genomes, except BCG Tice and Montreal sub-strains. A 20.3 kb prophage of *M. bovis* was replaced with 11.2 kb prophage in the “early” sub-strains genomes (BCG Tokyo, Moreau, Russia), but the former prophage was lost in “late” sub-strains genomes. The second group comprised six BCG Montreal prophages, and the third – 15 BCG Tice prophages. The prophages in these groups were specific and did not coincide with prophages of other sub-strains (see Fig. 6, circle B). The most of phage ORFs in the common *M. bovis* prophages were annotated as genes of Caudovirales (Myoviridae, Siphoviridae, Podoviridae) – typical bacteriophages. However, the most of phage ORFs in BCG Tice or BCG Montreal prophages were similar to the genes of various Herpes viruses (Human, Bovine, Macaci, Alcela, Anguil).

Comparative prophage analyses confirmed the mosaic BCG genome structure. Alignment of BCG Montreal and BCG Tice phage sequences with BCG Russia 368 complete genome indentified a high similarity only for fragments of BCG Tice/BCG Montreal prohages. They are shown in Fig. 6 (circle B) as the purple (BCG Tice) and blue (BCG Montreal) bars. Thus, 13.4 and 13.9 kb prophage of BCG Tice were subdivided into 5 and 3 fragments, respectively, in BCG Russia 368 genome. The comparison demonstrated multiple gaps ranging from 14 to 128 bp in the sequences of BCG Russia 368 that are homologous to BCG Montreal prophages.

In contrast, the alignment of 7.5 kb BCG Russia 368 prophage versus BCG Tice and Montreal genomes demonstrated that 0.9 and 6.6 kb fragments were residing in different regions of BCG Tice/ Montreal genomes. The transposase gene sequence from 7.5 kb BCG Russia 368 prophage was absent in BCG Tice / Montreal genomes.

Moreover, 7.5 kb BCG Russia 368 prophages sequence had 922 bp repeat in BCG Russia 368 genome (marked as red bar in Fug. 6, circle B). This sequence located in the same region as insertion element ISMt1. It should be noted that not just a fragment, but two whole prophages predicted by PHAST in BCG Russia 368 genome were also associated with the incretion elements. Thus, 7.5 kb prophage sequence was associated with IS1560 and 11 kb - with IS6110. The correlation between locations of the prophage sequences and the IS elements emphasizes a considerable contribution made by phages to the BCG genome evolution.

### Phylogeny reconstruction

**Fig. 8:**
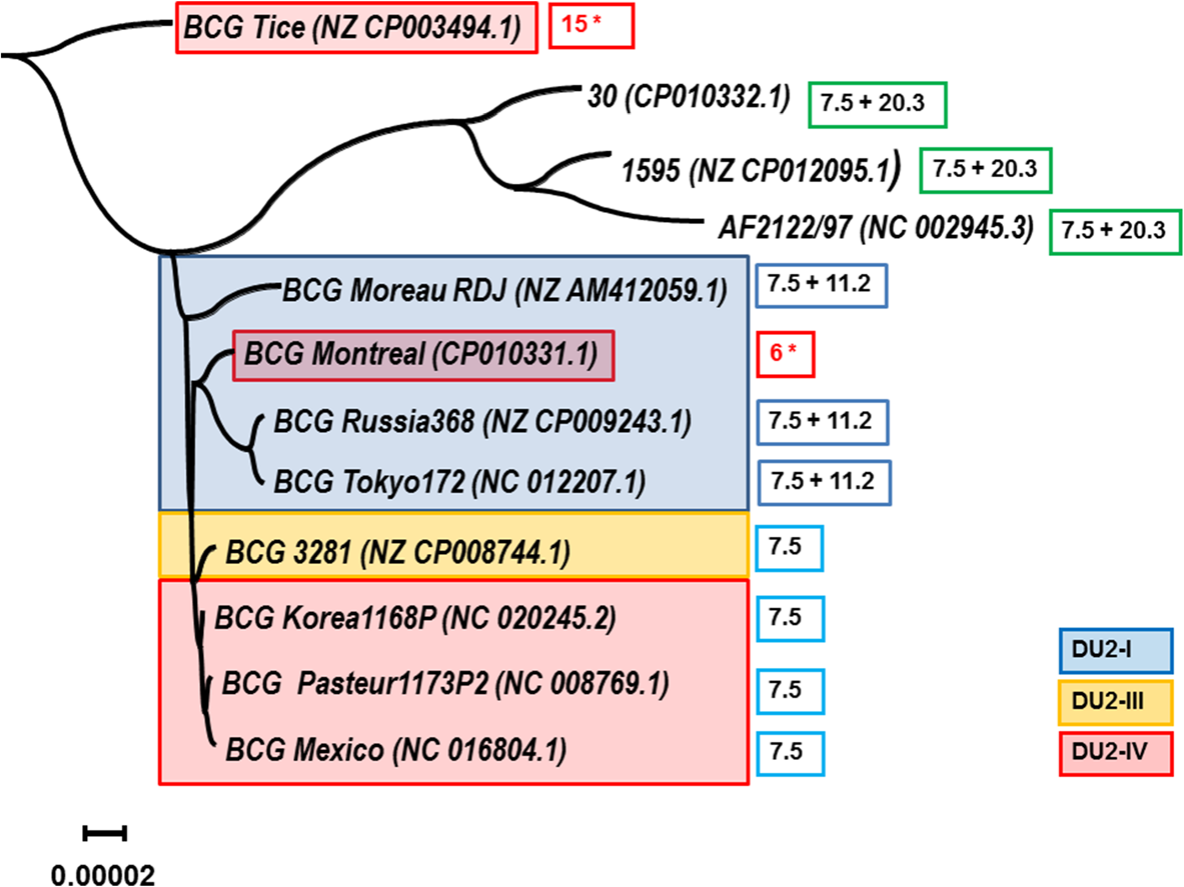
Phylogenetic tree for *M. bovis* strains based on full genome comparison with Neighbor Joining algorithm. Three colors of rectangles correspond to the type of DU2 region in BCG sub-strains: *blue* - DU2-I, *yellow* - DU2-III, red - DU2-IV. Sets of common prophages are indicated by colored frames. *Green*, *blue*, *dark blue* frames indicate prophage size in kb. *Red* frames with figures and asterisks reflect the number of prophages identified in BCG Tice and BCG Montreal genomes

Phylogeny reconstruction was made using the genome sequences of analyzed *M. bovis* strains and BCG sub-strains. The tree demonstrated in Fig. 8 was compared with known genealogical data of vaccine sub-strains based on DU2 region [38, 39] and with prophage profile. BCG sub-strains and *M. bovis* strains fall in two separate clades. In BCG clade the “early” (Russia, Tokyo) and the “late” sub-strains (Pasteur 1173P2, Korea 1168P, Mexico) have formed discrete closely related groups with a few exceptions. BCG Moreau sub-strain has formed a basal branch. The most outstanding discrepancies were associated with positions of BCG Tice and BCG Montreal. BCG Tice took the most divergent place on the tree and BCG Montreal unexpectedly fell into the group of the “early” sub-strains. BCG 3281, isolated from an adult patient with pulmonary tuberculosis, was close to the “late” sub-strains. Interestingly, each of group on the tree had specific sets of prophage sequences.

DU2 region genealogy correlated with the whole genome phylogeny in DU2-I “early” sub-strain group. BCG 3281 represented DU2-III group [40] creating a separate branch on the phylogenetic tree. But DU2-IV group was heterogeneous, since it included the above mentioned sub-strains BCG Tice and BCG Montreal. The discrepancy could be explained by the genome rearrangements caused by numerous prophage sequences.

## Discussion

The genomic variability of BCG sub-strains arose from one progenitor has been shown in multiple studies using comparative genome analysis. Identification of RDs, ins/del differences and SNPs indicates the continuous nature of in vitro evolution, which is still going on in BCG sub-strains. We assumed that prophages can contribute to genomic evolution and the BCG sub-strains diversity. Prophages are known to constitute as much as 10–20% of a bacterium genome. Many of these prophages appear to be defective and are in a state of mutational decay. However, recombination has occurred between related prophages that reside at different locations in a bacterium genome. In addition, many genes in defective prophages remain functional [41]. So prophages, including defective ones, can be important biological cause of genomic rearrangements. The analysis of these prophages revealed unexpected evolutionary patterns suggesting widespread contribution of prophages to bacterial fitness [42].

The comparative genome analysis of nine BCG sub-strains and three *M. bovis* strains suggested our assumption and revealed remarkable differences between their prophage profiles. On the other hand, unexpected changes in the genomes associated with the number and composition of prophages were discovered in the genomes of the late strains Tice and Montreal.

Both BCG Tice and BCG Montreal or Frappier were taken from the Pasteur Institute after 1934 and, according to the Brosch et al., they had close phylogenetic relations because they fall in one phylogenetic group “DU2 IV, Δint”[38]. The analysis of the history of BCG Tice sub-strain demonstrated that the first Tice sub-strain received by Dr. Rosenthal from the Pasteur Institute was a progenitor of at least six different daughter BCG sub-strains: H, K, E, L, LH, and BL. This fact emphasizes the heterogeneity of the “late” BCG sub-strains. In 1952 the sub-strain BL, shown to be strongly attenuated in laboratory studies, was mixed in the ratio 3:1 with a new routine ‘P’ strain, received from the Pasteur Institute in 1951. This new sub-strain, called BLP, was freeze-dried in 1952, and since 1953 only freeze-dried BCG vaccine from this mixed strain has been produced [33]. The history of BCG Montreal sub-strain is also known completely, because three times BCG sub-strains were sent to Canada from the Pasteur Institute [33].

The appearance of new prophage profiles in BCG Tice and BCG Montreal sub-strains reflects substantial changes of BCG genomes, which can also affect vaccine properties of the sub-strains. According to Zhang et al. [43] BCG Montreal/Frappier and BCG Tice along with BCG Phipps, BCG Prague sub-strains have lost the largest number of T-Cell epitopes, associated with its vaccine properties. In contrast, BCG Russia and BCG Tokyo sub-strains are still characterized by the largest number of T-Cell epitopes among other BCGs.

These findings prove the need for extensive genomic regions sequencing to identify prophages as potential markers of genomic rearrangement. Prophage studies allow better understanding of the genetic differences and characteristics of various BCG sub-strains and may also be useful for monitoring genetic stability of the seed lot sub-strain.

## Conclusions

The 21st century has been marked by the growth of human migration from the regions with high TB incidence and the increase in number of HIV-infected individuals. As a result, the emphasis in TB vaccination campaign has been shifted from children to adolescents and adults.

Fifteen vaccine candidates were assessed in clinical trials in 2015. They were designed either for BCG replacement vaccine or as a potential boost vaccine for the protection of adolescents and adults. The list included recombinant BCGs, attenuated *M. tuberculosis* strains, recombinant viral-vectored platforms, protein/adjuvant combinations, and mycobacterial extracts [1]. A subunit vaccine based on the mycobacterial proteins fused to cellulose-binding domain was developed in N.F. Gamaleya Research Center [44].

On the other hand, new area of BCG vaccine application has been proposed. As most humans are born in bacteriological environments characterized by a low microbial diversity, the effects of BCG vaccine administrated immediately after birth, as a modulator of Th-1/Th-2 responses, is very important and should be analyzed [45].

In this context, the task of BCG genome stability control is crucial and will continue to be relevant.

## Additional files

Additional file 1: Table S1. Primers used for BCG Russia 368 genome gaps closure by PCR and Sanger sequencing. (XLSX 14 kb)
Additional file 2: Table S2. Localization and description of mutation in BCG Russia 368 genome vs. BCG Tokyo 172. (XLSX 17 kb)
Additional file 3: Table S3. Localization of specific genome elements in BCG Russia genome. (XLSX 23 kb)

## Abbreviations

BCG: Bacille-Calmette-Guérin vaccine
ECBS: WHO Expert Committee on Biological Standardization
IS: Insertion sequences elements
ORF: Open reading frame
RD: Region of differences
TB: Tuberculosis
WGM: Whole genome map
WHO: World Health Organization
